# Correction to “Effect of vitamin D supplementation on circulating level of autophagosome protein LC3A, inflammation, and physical performance in knee osteoarthritis”

**DOI:** 10.1111/cts.13856

**Published:** 2024-05-29

Saengsiwaritt W , Jittikoon J , Chaikledkaew U , Tawonsawatruk T , Honsawek S , Udomsinprasert W . Effect of vitamin D supplementation on circulating level of autophagosome protein LC3A, inflammation, and physical performance in knee osteoarthritis. Clin. Transl. Sci. 2023;16(12):2543–2556.37749758 10.1111/cts.13646PMC10719460

In the first paragraph of the “Methods” section, the text “IRB No. MU‐DT/PY‐IRB2022PY072” was incorrect. The correct IRB number is “COA.No.MU‐DT/PY‐IRB 2022/032.3006”.

We apologize for this error.

